# Gene Variants of the OAS/RNase L Pathway and Their Association with Severity of Symptoms and Outcome of SARS-CoV-2 Infection

**DOI:** 10.3390/jpm14040426

**Published:** 2024-04-17

**Authors:** Aurelio Perez-Favila, Sonia Sanchez-Macias, Sergio A. Oropeza De Lara, Idalia Garza-Veloz, Roxana Araujo-Espino, Maria E. Castañeda-Lopez, Alejandro Mauricio-Gonzalez, Sodel Vazquez-Reyes, Perla Velasco-Elizondo, Perla M. Trejo-Ortiz, Fabiana E. Mollinedo Montaño, Claudia Castruita-De la Rosa, Margarita L. Martinez-Fierro

**Affiliations:** 1 Laboratorio de Medicina Molecular, Unidad Académica de Medicina Humana y Ciencias de la Salud, Universidad Autónoma de Zacatecas, Zacatecas 98160, Mexico; aurelio.perezf@uaz.edu.mx (A.P.-F.); 34156087@uaz.edu.mx (S.S.-M.); sergioantoniooropeza@gmail.com (S.A.O.D.L.); idaliagv@uaz.edu.mx (I.G.-V.); castruitade@gmail.com (C.C.-D.l.R.); 2 Doctorado en Ciencias con Orientación en Medicina Molecular, Unidad Académica de Medicina Humana y Ciencias de la Salud, Universidad Autónoma de Zacatecas, Zacatecas 98160, Mexico; roxana.araujo@uaz.edu.mx (R.A.-E.); maria.cl@uaz.edu.mx (M.E.C.-L.); amgdark@uaz.edu.mx (A.M.-G.); vazquezs@uaz.edu.mx (S.V.-R.); pvelasco@uaz.edu.mx (P.V.-E.); perlatrejo@uaz.edu.mx (P.M.T.-O.); fabiana.mollinedo@uaz.edu.mx (F.E.M.M.)

**Keywords:** COVID-19, SARS-CoV-2, coronavirus, gene variant, *RNASEL*, OAS, polymorphism

## Abstract

Introduction: The interferon pathway plays a critical role in triggering the immune response to SARS-CoV-2, and these gene variants may be involved in the severity of COVID-19. This study aimed to analyze the frequency of three gene variants of *OAS* and *RNASEL* with the occurrence of COVID-19 symptoms and disease outcome. Methods: This cross-sectional study included 104 patients with SARS-CoV-2 infection, of which 34 were asymptomatic COVID-19, and 70 were symptomatic cases. The variants rs486907 (*RNASEL*), rs10774671 (*OAS1*), rs1293767 (*OAS2*), and rs2285932 (*OAS3*) were screened and discriminated using a predesigned 5′-nuclease assay with TaqMan probes. Results: Patients with the allele C of the *OAS2* gene rs1293767 (OR = 0.36, 95% CI: 0.15–0.83, *p* = 0.014) and allele T of the *OAS3* gene rs2285932 (OR = 0.39, 95% CI: 0.2–0.023, *p* = 0.023) have lower susceptibility to developing symptomatic COVID-19. The genotype frequencies (G/G, G/C, and C/C) of rs1293767 for that comparison were 64.7%, 29.4%, and 5.9% in the asymptomatic group and 95.2%, 4.8%, and 0% in severe disease (*p* < 0.05). Conclusions: Our data indicate that individuals carrying the C allele of the *OAS2* gene rs1293767 and the T allele of the *OAS3* gene rs2285932 are less likely to develop symptomatic COVID-19, suggesting these genetic variations may confer a protective effect among the Mexican study population. Furthermore, the observed differences in genotype frequencies between asymptomatic individuals and those with severe disease emphasize the potential of these variants as markers for disease severity. These insights enhance our understanding of the genetic factors that may influence the course of COVID-19 and underscore the potential for genetic screening in identifying individuals at increased risk for severe disease outcomes.

## 1. Introduction

Severe acute respiratory syndrome coronavirus 2 (SARS-CoV-2) is a highly contagious and virulent virus, and the third coronavirus to cause a pandemic [1]. The other two previous coronaviruses were severe acute respiratory syndrome coronavirus (SARS-CoV) and Middle East respiratory syndrome coronavirus (MERS-CoV) [2]. The global pandemic caused by SARS-CoV-2 was officially declared by the World Health Organization (WHO) on 11 March 2020 [3]. As of 20 February 2024, 7,028,881 deaths and 774,593,066 cases have been reported worldwide, while 334,958 deaths and 7,702,809 cases have been reported in Mexico [4].

Human-to-human transmission of SARS-CoV-2 occurs by several routes, including direct transmission, contact transmission, airborne transmission through aerosols, and during medical procedures [5]. The most common clinical manifestations are fever, cough, shortness of breath, muscle aches, confusion, headache, sore throat, rhinorrhea, chest pain, diarrhea, and nausea; however, these manifestations vary greatly among patients [6].

SARS-CoV-2 binds to angiotensin-converting enzyme 2 (ACE2) receptors and their co-receptor, transmembrane protease serine 2 (TMPRSS2), via the spike protein [7]. The SARS-CoV-2 membrane fuses with the host cell membrane and penetrates by endocytosis, after that, the nucleocapsid is released into the cytosol [1]. Subsequently, the viral RNA genome is translated into the replicate proteins [1]. Toll-like receptors (TLR3 and TLR7) and cytosolic RNA sensors (RIG-I: retinoic acid-inducible gene I and MDA-5: melanoma differentiation-associated protein 5) are involved in the first steps of the cellular response to viral infection, recognizing the replication intermediates and viral RNA of SARS-CoV-2 [8]. Subsequently, the transcription factors nuclear factor κappa B (NFκB), interferon regulatory factor (IRF) 3 (IRF3), and 7 (IRF7) are activated via the tumor necrosis factor receptor-associated factor 6 (TRAF6)/IKKα/β/γ and tumor necrosis factor receptor-associated factor 3 (TRAF3)-TBK1/IKKε pathways, respectively, and the production of both interferons (type I and type III) begins [8]. The binding of IFNs to cellular receptors promotes the transcription of hundreds of interferon-stimulated genes (ISGs) [8]. Among the activated genes is the 2′–5′-oligoadenylate synthetase (*OAS*) family, which has significant antiviral functions [9]. OAS1, OAS2, OAS3, and OAS-like proteins are four types of genes belonging to the *OAS* family [10]. For ribonuclease L (RNase L) homodimerization and activation to occur, the OAS enzyme must bind to viral dsRNA that synthesizes 2–5′-oligoadenylate from ATP, which is generally expressed in an inactive form [8]. RNase L has several mechanisms of antiviral activity, some including degradation of the viral genome, degradation of viral mRNA, degradation of cellular mRNA and rRNA, and enhancement of IFN signaling [11].

There are studies focusing on evaluating the different gene variants of the *OAS* family and their effects on the host antiviral response. Some of them are associated with a differential immune response, such as the A allele of the rs10774671 variant (*OAS1*), which has been associated with a less effective immune response against RNA virus infections due to the reduced activity of this isoform of OAS1 [12]. Another study conducted in 638 Caucasian patients with self-limiting and persistent hepatitis C virus (HCV) infection showed an association between a gene variant in the 3′-untranslated region (3′UTR) of the *OAS1* gene and self-limiting HCV infection [13]. A case-control study about gene variants in the 3′-UTR of *OAS1* correlated with SARS infection showed that the A/G and G/G have a more substantial protective effect against SARS than the A/A genotype [14].

Although the outcome of COVID-19 can sometimes be explained by the presence of comorbidities such as diabetes, hypertension, obesity, and chronic kidney disease [15], there is a significant proportion of patients in whom clinical symptoms vary widely, and the risk of severe disease is not explained by clinical risk factors, suggesting the existence of a genetic predisposition that influences the response to SARS-CoV-2 [16]. Type I interferons and III participate in the modulation of the innate immune system during viral infection [8]. The role of different genetic variants of the *OAS* gene family in viral diseases and their development has been studied, but their role in patients with COVID-19 is poorly known [12,13]. In this study, we analyzed the frequency of three gene variants of *OAS* (*OAS1*, *OAS2*, and *OAS3*) and one in *RNASEL*, and evaluated their association with the occurrence of COVID-19 symptoms and disease outcome.

## 2. Materials and Methods

### 2.1. Study Population and Selection Criteria

The Committee of the Academic Unit of Human Medicine and Health Sciences of the Universidad Autónoma de Zacatecas and the Ethics and Investigation Committee of the Hospital General de Zacatecas Luz González Cosío (ID numbers: 0223/2021/C, AMCCI-FSARSC2-006, and AMCCI-FSARSC2-007) approved this cross-sectional study. All patients provided written informed consent prior to participation.

We enrolled 104 subjects with indicated screening for SARS-CoV-2. Mexican patients older than 18 years who lived in Zacatecas state were eligible to participate. There were no exclusion criteria for the study. The recruitment of patients with mild COVID-19 and COVID-19 asymptomatic controls was carried out in the facilities of the Molecular Medicine Laboratory at Universidad Autónoma de Zacatecas. Patients with positive tests for SARS-CoV-2 with moderate and/or severe COVID-19 who required hospitalization [17] at the Hospital General de Zacatecas Luz González Cosío were also included. After recruitment, the patients were divided into two groups: the first group consisted of individuals with confirmed SARS-CoV-2 infection by a positive quantitative reverse transcription polymerase chain reaction (qRT-PCR) test and mild to severe disease; the second group consisted of patients with SARS-CoV-2 infection confirmed by a positive qRT-PCR test but asymptomatic. The study involved 70 patients with COVID-19 who presented symptoms and 34 patients with asymptomatic COVID-19.

### 2.2. Medical Data Collection and Parameters for the Categorizing of Patients Based on the Severity of COVID-19

For hospitalized patients, on the first day of hospital admission, the patient underwent a standardized medical examination, which included a thorough evaluation of the medical history, lifestyle, and medication intake. The medical professionals compiled the subsequent healthcare metrics to categorize patients: temperature, anthropometric characteristics, heart and respiratory rates, blood pressure, and oxygen saturation (SaO_2_). Laboratory data from the clinical records included the following: hemoglobin, urea, blood urea nitrogen, glucose, Dimer D, erythrocyte sedimentation rate, lactate dehydrogenase, C-reactive protein, and procalcitonin. On the other hand, after their inclusion in the study, patients with COVID-19 were subclassified as mild, moderate, severe, and critical diseases in conformity with the COVID-19 disease severity classification of the WHO [18].

### 2.3. SARS-CoV-2 Screening

Two swabs were taken from each participant, one from the nasopharyngeal and the other from the oropharyngeal. Subsequently, the samples were packed and transported in triple packaging at a temperature of 4 °C following the WHO and the Pan-American Health Organization guidelines for handling and transporting viral SARS-CoV-2 specimens [19,20]. Briefly, the samples were processed using the CDC qRT-PCR panel (Integrated DNA Technologies, Coralville, IA, USA), and the presence/absence was detected in Step One Plus Real-time PCR system (Thermos Fisher Scientific, Waltham, MA, USA) [21] in the Molecular Medicine Laboratory at Zacatecas.

### 2.4. DNA Isolation and Genotyping

Each patient donned 2 mL of a peripheral blood sample collected in a standard tube with anticoagulant (EDTA). After collecting blood samples, we extracted the DNA. A total of 500 μL of peripheral blood samples were used to obtain total genomic DNA using a standard phenol/chloroform technique. To obtain the DNA concentration, we employed a NanoDrop-1000 spectrophotometer (Thermo Scientific, Waltham, MA, USA). DNA samples were stored at −20 °C. The gene variants of interest [(rs10774671 (*OAS1*: ID_ 4938), rs1293767 (*OAS2*: ID_4939), rs2285932 (*OAS3*: ID_4940), and rs486907 (*RNASEL*: ID_6041)], were evaluated by employing a predesigned 5′-nuclease assay with TaqMan MGB probes (Applied Biosystems: Foster City, CA, USA). The reactions were carried out using 10 μL of solution and 10 ng of genomic DNA, using standard PCR cycling conditions. Allele discrimination was performed using the StepOne RT-PCR system with the StepOne v2.0 software (Applied Biosystems).

### 2.5. Statistical Methods

We executed the statistical analyses by employing Sigma Plot v14.5 software. To present the results, the mean ± standard deviation (SD) or the percentage (%) was obtained. The general variables of the study such as age, sex, oxygen saturation, comorbidities, and biochemical variables were compared using the chi-square test or student’s *t*-test. The Hardy–Weinberg (HW) test was carried out to detect HW equilibrium deviations for each gene variant. The allele, genotype, and haplotype frequencies and their comparisons between the study groups were calculated using the SNPStats software https://www.snpstats.net/ accessed on 1 June 2023 [22]. The odds ratio (OR) values with a 95% confidence interval (CI) were obtained for all the comparisons. Statistical significance was determined by using a two-sided significance level of *p* < 0.05.

## 3. Results

### 3.1. General Findings and Comparisons of the Genotype/allele Frequencies of the Gene Variants of the OAS/RNASEL Pathway between Asymptomatic and Symptomatic Groups with COVID-19

The study enrolled 104 Mexican patients, 70 with symptomatic COVID-19 and the remainder with asymptomatic COVID-19. The mean age of the symptomatic patients with COVID-19 was 58.3 ± 14.6 years, and 50.5 ± 18.3 years was the average age of the asymptomatic controls (*p* = 0.027). In total, 38 (54.3%) of the symptomatic patients with COVID-19 and 24 (70.6%) of the asymptomatic controls were men (*p* = 0.112). The COVID-19 severity was distributed as follows: 12 (11.5%) patients had mild disease, 21 (20.2%) moderate disease, 21 (20.2%) severe disease, and critical disease was observed in 16 (15.4%) participants. The main clinical findings in the symptomatic group were dyspnea (64.3%), fever (60%), tachypnea (57.1%), headache (48.6%), myalgia (47.1%), and cough (45.7%), (Table 1).

All the gene variants included in the study were in HW equilibrium (*p* > 0.05). Table 2 shows the analysis of allele and genotype frequencies in symptomatic and asymptomatic groups. The frequencies for the C/C, C/T, and T/T genotypes of the rs486907 (*RNASEL*) variant were 62.9%, 31.4%, and 5.7% in the symptomatic group, and 70.6%, 23.5%, and 5.9% in the asymptomatic controls. For the variant rs10774671 (*OAS1*), the frequency of genotypes A/A, A/G, and G/G was 97.1%, 2.9%, and 0% in the symptomatic patients and 94.1%, 5.9% and 0% in the asymptomatic group, respectively. No significant association was found between genotypes of rs486907 (*RNASEL*) or for the rs10774671 (*OAS1*) variant and symptomatic COVID-19 (*p* > 0.05). In the same way, the distribution of frequencies of the minor allele both for the rs486907 (T allele) and rs10774671 (G allele) variants showed no differences between groups of asymptomatic and asymptomatic patients with COVID-19 (*p* > 0.05).

For the gene variant *OAS2* rs1293767, the frequencies found for the G/G, G/C, and C/C genotypes were as follows: 82.9%, 17.1%, and 0% for the symptomatic group and 64.7%, 29.4%, and 5.9% for the asymptomatic group, (*p* = 0.03), respectively. The patients with the C/G genotype of rs1293767 showed decreased odds of developing symptomatic COVID-19 (OR = 0.46, 95% CI: 0.17–1.20; *p* = 0.03). The proportion for the minor allele (C allele) was 9% in the symptomatic group and 21% in the asymptomatic group. Carriers of the C allele were less likely to be developing symptomatic COVID-19 (OR = 0.36, 95% CI: 0.15–0.83; *p* = 0.014).

The distribution of the genotype frequencies of the *OAS3* rs2285932 variant in the study groups showed that the genotypes C/C, C/T, and T/T were present in 81.4%, 18.6%, and 0% of the participants in the symptomatic group, and in 64.7%, 29.4%, and 5.9% in the asymptomatic patients with COVID-19 (*p* = 0.039). Heterozygous for the rs2285932 variant had decreased odds of symptomatic COVID-19 (OR = 0.50, 95% CI: 0.19–1.31; *p* = 0.039). Similarly, the proportion of the minor allele (T allele) for this gene variant was 9% in the symptomatic group and 21% in the asymptomatic group. Individuals with one allele T of rs2285932 had decreased odds of developing symptomatic COVID-19 (OR = 0.39, 95% CI: 0.2–0.9; *p* = 0.023).

### 3.2. Comparisons of Genotype/Allele Frequencies of the Gene Variants of OAS/RNASEL Pathway between Hospitalized and Non-Hospitalized Patients with COVID-19

During the study, 52 patients required hospitalization, and 52 did not. Table 3 shows the general characteristics of the hospitalized and non-hospitalized patients. The mean age of patients hospitalized because of COVID-19 was 58.3 ± 14.3 years, and the mean age of patients not hospitalized was 51.1 ± 17.9 years (*p* = 0.042). Significant differences (*p* < 0.05) in the following symptoms were found between hospitalized and non-hospitalized groups: chest pain (13.5% vs. 34.6%), asthenia (32.7% vs. 0%), adynamia (32.7% vs. 0%), anosmia (3.8% vs. 30.8%), and dysgeusia (7.7% vs. 26.9%).

Table 4 shows the results of the comparison of allele and genotype frequencies between patients with COVID-19 who are non-hospitalized and hospitalized. The frequency of minor alleles of rs486907, rs10774671, rs1293767, and rs2285932 were 22%, 4.0%, 15%, and 15% for non-hospitalized patients, and 18%, 4%, 10%, and 11% for hospitalized patients, respectively. There were no differences in the allele or genotype proportions of the interest variants between hospitalized and non-hospitalized patients (*p* > 0.05).

### 3.3. Gene Variants of OAS/RNase L Pathway and COVID-19 Severity

To evaluate the associations between the gene variants of interest and the severity of COVID-19, the patients in the protocol were classified as asymptomatic, mild, moderate, severe, and critical disease. Table 5 displays a summary of comparisons carried out to identify associations between the gene variants included in the study and the severity of COVID-19. These analyses showed differences only in proportions of the rs1293767 (*OAS2*) variant between asymptomatic and severe COVID-19. The genotype frequencies (G/G, G/C, and C/C) of rs1293767 for that comparison were 64.7%, 29.4%, and 5.9% in the asymptomatic group and 95.2%, 4.8%, and 0% in severe disease (*p* < 0.05). Considering the patients with asymptomatic COVID-19 as a reference, there were no differences between genotype proportions of rs486907, rs10774671, and rs2285932 and mild, moderate, severe, or critical COVID-19 (*p* > 0.05).

These analyses showed differences only in proportions of the rs1293767 (*OAS2*) variant between asymptomatic and severe COVID-19 (Table 5). The genotype frequencies (G/G, G/C, and C/C) of rs1293767 for that comparison were 64.7%, 29.4%, and 5.9% in the asymptomatic group and 95.2%, 4.8%, and 0% in severe disease (*p* < 0.05). Considering the patients with asymptomatic COVID-19 as a reference, there were no differences between genotype proportions of rs486907, rs10774671, and rs2285932 and mild, moderate, severe, or critical COVID-19 (*p* > 0.05).

### 3.4. Gene Variants of OAS/RNase L Pathway and COVID-19 Outcome

To evaluate whether there was an association between the outcome of COVID-19 and the gene variants included in the study, the hospitalized patients were classified according to their outcome as those who did not survive COVID-19 and those who recovered. Table 6 displays the laboratory parameters and comorbidities of hospitalized patients with COVID-19 classified according to their outcome.

The predominant coexisting conditions were type 2 diabetes mellitus (T2DM), obesity, and hypertension. Hypertension was most frequent in patients who did not survive COVID-19 (62.96%) compared to those who recovered (28%) with a statistical difference (*p* = 0.012). The laboratory findings show that blood glucose was elevated at admission (178.92 mg/dL ± 75.8) and discharge (184.07 mg/dL ± 103.74) in hospitalized patients who did not survive COVID-19. Also, the glucose of the hospitalized patients who recovered was higher on admission (192.50 mg/dL ± 144.72) than at discharge (140.42 mg/dL ± 65.38). Blood chemistry showed that hospitalized patients who did not survive COVID-19 had elevated levels of triglycerides, creatinine, blood urea nitrogen, C-reactive protein, and procalcitonin compared to those who recovered but without statistical differences. In the biometric assessment of hospitalized patients who did not survive COVID-19, there was leukocytosis with neutrophilia and lymphopenia.

Table 7 shows the results obtained from the comparisons of the genotypes/alleles between the groups of patients according to their outcomes. Associations between the gene variants evaluated in the study and the outcome of COVID-19 were not identified (*p* > 0.05).

### 3.5. Haplotype Analyses of the Gene Variants of the OAS/RNase L Pathway and Their Association with the COVID-19 Symptoms, Severity, and Outcome

The results of the haplotype comparison of the gene variants rs486907 (*RNASEL*), rs10774671 (*OAS1*), rs1293767 (*OAS2*), and rs2285932 (*OAS3*) between the different study groups are shown in Table 8, Table 9 and Table 10. There was no association between the presence/absence of COVID-19 symptoms and the haplotypes evaluated (*p* > 0.05; Table 8). Considering the haplotype frequencies between non-hospitalized and hospitalized patients, there was a rare haplotype TGCT with a frequency of 2.4% with a *p* < 0.0001. Similarly, in recovered patients and patients who did not survive COVID-19, a rare haplotype CGCT (0.9%) was found with a *p* < 0.0001. Both significant haplotypes increased the odds of hospitalization and death because of COVID-19 (Table 9 and Table 10).

## 4. Discussion

Given the recent onset of COVID-19, there are still many questions about the genetic susceptibility to the disease. Differences in COVID-19 mortality rates are evident across various countries, with recorded extremes such as a high infant mortality rate of 2.56% in Africa and a low case fatality rate (CFR) of 0.79% in Australia [23]. These disparities are likely influenced by an array of population-specific characteristics that affect disease outcomes. These factors encompass prevalent comorbidities, including coronary heart disease, diabetes, and hypertension [24], and genetic determinants that are not yet fully elucidated but are believed to play a role in disease pathogenesis. Furthermore, evidence suggests COVID-19 disproportionately impacts different populations, affecting susceptibility, disease severity, and rates of hospitalization. However, the nature of these disparities is not completely understood, highlighting the need for further research to unravel the mechanisms of disease [25]. Genetic variants could explain the variability in the clinical presentation of patients with COVID-19; some of them are found in *ACE2*, *TMPRSS2*, and the *OAS* family gene [26]. In this study, we examined the frequency of three gene variants within the *OAS* family (*OAS1*, *OAS2*, and *OAS3*) and one in *RNASEL*, in relation to the manifestation of COVID-19 symptoms and the overall disease prognosis.

Our findings indicate that individuals possessing the C/G genotype of the OAS2 gene variant rs1293767 have 60% lower odds (or a reduction in risk by 60%) of experiencing severe COVID-19 symptoms. Furthermore, individuals with the C allele of the same gene variant (rs1293767 in *OAS2*) exhibit a 70% decrease in the likelihood of developing any COVID-19 symptoms. Similarly, in a previous study in the Mexican population, the genetic variant *OAS2* rs1293767 was associated with COVID-19 infection and severe disease in the general population and in patients with COVID-19 [27]. The highest molecular weight isoform of the *OAS* family is OAS3, whose expression is activated by type 1 and type 3 interferon [28]. In our study, individuals carrying the heterozygous C/T genotype of the *OAS3* gene variant rs2285932 were at a 50% reduced risk of developing COVID-19 symptoms compared to those without this genotype (OR = 0.50; 95% CI: 0.19–1.31). Additionally, individuals with the T allele of the gene variant rs2285932 (OAS3) had a 61% lower chance of exhibiting COVID-19 symptoms (OR = 0.39; 95% CI: 0.2–0.9). The role of the rs2285932 (*OAS3*) gene variant in the context of SARS-CoV-2 infection has been relatively underexplored, making our findings particularly significant [29]. In the study conducted by Chaaithanya et al., the authors examined the relationship between various polymorphisms and CHIKV (Chikungunya virus) infection, which included the rs2285932 variant in the *OAS3* gene. They discovered that the *OAS* gene family influences the likelihood of clinical symptom development in patients infected with CHIKV. This finding is akin to what our study reveals about the role of *OAS* genes in determining the severity of symptoms in patients with SARS-CoV-2 infection, despite the difference in the etiological agents involved [29]. OAS1 has plausible biological activity against SARS-CoV-2 by participating in the innate immune response against SARS-CoV-2 viruses [26]. In this study, we observed no significant correlation between the *OAS1* rs10774671 variant and an elevated risk of developing severe symptoms caused by SARS-CoV-2 infection. In contrast, a previous study focused on pediatric patients reported that those with the G/C genotype of *OAS1* exhibited a 0.18-fold reduction in the risk of COVID-19, suggesting a protective effect. On the other hand, the presence of the A allele was associated with a 5.7-fold increase in the risk of more severe disease outcomes in COVID-19 [30]. These apparent divergences may be associated with differences between study designs and/or differences between the populations evaluated; however, it is important to consider that, in our study, the limited sample size impacted the representativeness of the G/G genotype during the analysis of the genetic variant *OAS1* rs10774671, which could have introduced biases in the statistical analysis. Therefore, the lack of association of *OAS1* rs10774671 and COVID-19 susceptibility, severity and/or outcome should be considered with caution.

The *RNASEL* gene is an indispensable part of the interferon-mediated immune response to the virus, located on chromosome 1q25 and encoding ribonuclease L [31]. In our research, we detected no significant link between the rs486907 (*RNASEL*) gene variant and the onset of COVID-19 symptoms. The literature on the *RNASEL* rs486907 variant and its connection to SARS-CoV-2 is limited. A case-control study that investigated the correlation between the *RNASEL* rs486907 variant and the presence of various viruses in prostate tissue did not reveal any significant associations [32]. Nonetheless, in an interesting previous study, authors carried out whole-exome or whole-genome sequencing in children with multisystem inflammatory syndrome (MIS-C) from the international COVID Human Genetic Effort (CHGE) cohort. Authors identified the presence of autosomal recessive deficiencies of OAS1, OAS2, or RNASEL (*OAS1* p.R47*, *OAS2* p.R535Q, p.Q258L, and p.V290I, and *RNASEL* p.G59S + E265*) as genetic etiologies of MIS-C in 5 unrelated children. In these patients, MIS-C may result from an excessive response of immune cells to SARSCoV-2 dsRNA intermediates. RNase L/OAS1-2 genetic deficiency resulted in the immunological and clinical phenotype of MIS-C [33]. Although those genetic deficiencies are rare and were not assessed in our study or in other populations, their results provided a plausible pathogenic mechanism involving the RNase L/OAS1-2 pathway for MIS-C.

In this study, when analyzing the haplotype frequencies of the gene variants rs486907 (*RNASEL*), rs10774671 (*OAS1*), rs1293767 (*OAS2*), and rs2285932 (*OAS3*) among patients, significant differences were observed. Specifically, patients carrying the rare TGCT haplotype were found to have increased odds of requiring hospitalization due to SARS-CoV-2 infection. Similarly, when comparing patients who recovered to those who succumbed to the infection, the rare CGCT haplotype was associated with a heightened risk of mortality from SARS-CoV-2. The genetic variants analyzed in our study in relation to SARS-CoV-2 infection have not been extensively investigated; however, in a recent study, the *OAS1/OAS3* haplotype ‘GTTG’ carrying a functional allele G of splice-acceptor variant rs10774671 manifested its protective function in the Delta pandemic wave [34]. Another study highlighted that a Neanderthal-derived haplotype encompassing the genes *OAS1*, *OAS2*, and *OAS3* conferred protection against COVID-19, with *OAS3* showing the most substantial correlations [35]. Although the three studies are not entirely comparable, the positive associations found at the haplotype level involving the *OAS* genes are crucial in determining which variants play a role in modulating the immune response and the immunopathogenesis of viral infections. In Table 11, we present a comparative summary of the most relevant association studies, which evaluated the role of gene variants of the OAS/RNase L pathway and COVID-19.

The potential utility and applicability of our findings, particularly in detecting the rs1293767 *OAS2* and rs2285932 *OAS3* variants (in an individual manner) and/or the haplotype TGCT (*RNASEL* rs486907, *OAS1* rs10774671, *OAS2* rs1293767, *OAS3* rs2285932), are significant. They may assist in identifying individuals at a heightened risk of developing symptomatic COVID-19, as well as those who may suffer from its more severe consequences. Moreover, these insights have the potential to inform clinical decisions, guiding the treatment and management of patients by anticipating the trajectory of COVID-19. However, it is important to highlight, that in addition to the sample size, the homogeneity of the population in our study represents another study limitation, given that our cohort consisted of a Mexican mestizo population (a group resulting from the admixture of Native Americans, Spaniards, and Africans). Consequently, our findings may be most relevant for demographic groups with this genetic background. We recognize the need to replicate this study across a more ethnically and geographically diverse cohort to enhance the generalizability of our results.

## 5. Conclusions

The C allele and the C/G genotype of the rs1293767 *OAS2* genetic variant and the T allele and the C/T genotype of the rs2285932 genetic variant of *OAS3* had lower odds of developing COVID-19 symptoms among the studied Mexican population. Furthermore, the observed differences in genotype frequencies between asymptomatic individuals and those with severe disease emphasize the potential of these variants as markers for disease severity. These insights enhance our understanding of the genetic factors that may influence the course of COVID-19 and underscore the potential for genetic screening in identifying individuals at increased risk for severe disease outcomes. Future studies that include participants from varied ethnical backgrounds will be necessary for validating and extending the applicability of our findings to broader populations.

## Figures and Tables

**Table 1:** General characteristics of the study population.

Variable	Symptomatic COVID-19 Cases (*n* = 70)	Asymptomatic Controls (*n* = 34)
Sex *n* (%)		
Male	38 (54.3)	24 (70.6)
Female	32 (45.7)	10 (29.4)
Age, years (±SD)	58.3 ± 14.6	50.0 ± 18.3
Symptoms *n* (%)		
Fever	42 (60)	NA
Cough	32 (45.7)	NA
Cefalea	34 (48.6)	NA
Dyspnea	45 (64.3)	NA
Tachypnea	40 (57.1)	NA
Tachycardia	4 (5.7)	NA
Chest pain	25 (35.7)	NA
Vomit	5 (7.1)	NA
Diarrhea	16 (22.9)	NA
Asthenia	17 (24.3)	NA
Adynamia	17 (24.3)	NA
Myalgia	33 (47.1)	NA
Arthralgia	30 (42.9)	NA
Anosmia	18 (25.7)	NA
Dysgeusia	18 (25.7)	NA
Rhinorrhea	16 (22.8)	NA
Odynophagia	20 (28.6)	NA
General discomfort	30 (42.8)	NA

Data are represented as percentages. NA: Not applicable. SD: Standard deviation.

**Table 2:** Comparison of allele and genotype frequencies between the symptomatic and asymptomatic COVID-19 groups.

dbSNP ID	Genotype/Minor Allele	Symptomatic COVID-19 (*n* = 70)	Asymptomatic COVID-19 (*n* = 34)	OR (95% CI)	*p*-Value
*RNASEL* rs486907	C/C	44 (62.9)	24 (70.6)	1	0.7
	C/T	22 (31.4)	8 (23.5)	1.50 (0.58–3.88)	
	T/T	4 (5.7)	2 (5.9)	1.09 (0.19–6.40)	
	Allele T	30 (21.0)	12 (18.0)	1.27 (0.60–2.68)	0.524
*OAS1* rs10774671	A/A	68 (97.1)	32 (94.1)	1	0.47
	A/G	2 (2.9)	2 (5.9)	0.47 (0.06–3.49)	
	G/G	0 (0)	0 (0)	0.00 (0.00–NA)	
	Allele G	2 (1.0)	2 (3.0)	0.47 (0.065–3.47)	0.456
*OAS2* rs1293767	G/G	58 (82.9)	22 (64.7)	1	0.03 *
	G/C	12 (17.1)	10 (29.4)	0.46 (0.17–1.20)	
	C/C	0 (0)	2 (5.9)	0.00 (0.00–NA)	
	Allele C	12 (9.0)	14 (21.0)	0.36 (0.15–0.83)	0.014 *
*OAS3* rs2285932	C/C	57 (81.4)	22 (64.7)	1	0.039 *
	C/T	13 (18.6)	10 (29.4)	0.50 (0.19–1.31)	
	T/T	0 (0)	2 (5.9)	0.00 (0.00–NA)	
	Allele T	13 (9.0)	14 (21.0)	0.39 (0.2–0.9)	0.023 *

Abbreviations: CI, confidence interval; OR, odds ratio. Data are represented as frequency and percentages. * *p* < 0.05.

**Table 3:** General characteristics of patients hospitalized for COVID-19 and those not hospitalized.

Variable	Hospitalized (*n* = 52)	Non-Hospitalized (*n* = 52)	*p*-Value
Sex *n* (%)			
Male	24 (46.2)	36 (69.2)	0.013
Female	28 (53.8)	16 (30.8)	
Age (years)	58.3 ± 14.3	51.15 ± 17.9	0.0423 *
Symptoms *n* (%)			
Fever	23 (44.2)	19 (36.5)	0.602
Cough	19 (36.5)	13 (25)	0.323
Headache	13 (25)	21 (40.4)	0.222
Dyspnea	29 (55.8)	16 (30.8)	0.104
Tachypnea	13 (25)	11 (21.2)	0.713
Tachycardia	4 (7.7)	0 (0)	0.05
Chest pain	7 (13.5)	18 (34.6)	0.047 *
Vomit	2 (3.8)	3 (5.8)	0.072
Diarrhea	4 (7.7)	12 (22.9)	0.062
Asthenia	17 (32.7)	0 (0)	<0.001 *
Adynamia	17 (32.7)	0 (0)	<0.001 *
Myalgia	14 (26.9)	19 (36.5)	0.448
Arthralgia	12 (23.1)	18 (34.6)	0.334
Anosmia	2 (3.8)	16 (30.8)	0.002 *
Dysgeusia	4 (7.7)	14 (26.9)	0.029 *
Rhinorrhea	8 (15.4)	8 (15.4)	1
Odynophagia	7 (13.5)	13 (25)	0.219
General discomfort	10 (19.2)	20 (38.5)	0.107

Data are represented as frequency and percentages. * *p* < 0.05.

**Table 4:** Comparison of allele and genotype frequencies between patients with COVID-19 categorized as non-hospitalized and hospitalized.

dbSNP ID	Genotype/ Minor Allele	No Hospitalized (*n* = 52)	Hospitalized (*n* = 52)	OR (95%CI)	*p*-Value
*RNASEL* rs486907	C/C	32 (61.5)	36 (69.2)	1	0.68
	C/T	17 (32.7)	13 (25.0)	0.68 (0.29–1.61)	
	T/T	3 (5.8)	3 (5.8)	0.89 (0.17–4.72)	
	Allele T	23 (22.0)	19 (18.0)	0.78 (0.39–1.55)	0.49
*OAS1* rs10774671	A/A	50 (96.2)	50 (96.2)	1	1
	A/G	2 (3.8)	2 (3.8)	1.00 (0.14–7.38)	
	G/G	0 (0)	0 (0)	0 (NA)	
	Allele G	2 (4.0)	2 (4.0)	1	1
*OAS2* rs1293767	G/G	38 (73.1)	42 (80.8)	1	0.21
	G/C	12 (23.1)	10 (19.2)	0.75 (0.29–1.94)	
	C/C	2 (3.8)	0 (0)	0.00 (0.00–NA)	
	Allele C	16 (15.0)	10 (10.0)	0.58 (0.25–1.35)	0.208
*OAS3* rs2285932	C/C	38 (73.1)	41 (78.8)	1	0.23
	C/T	12 (23.1)	11 (21.1)	0.85 (0.34–2.15)	
	T/T	2 (3.8)	0 (0)	0.00 (0.00–NA)	
	Allele T	16 (15.0)	11 (11.0)	0.65 (0.28–1.47)	0.302

Abbreviations: CI, confidence interval; OR, odds ratio. Data are represented as frequency and percentages.

**Table 5:** Comparisons of the study groups.

Gene/Variant	COVID-19 Group Comparison			
	Asymptomatic (*n* = 34) versus:			
	Mild Disease (*n* = 12)	Moderate Disease (*n* = 21)	Severe Disease (*n* = 21)	Critical Disease (*n* = 16)
*RNASEL*/rs486907	NS	NS	NS	NS
*OAS1*/rs10774671	NS	NS	NS	NS
*OAS2*/rs1293767	NS	NS	*	NS
*OAS3*/rs2285932	NS	NS	NS	NS

NS: non-significant. * *p* < 0.05.

**Table 6:** Laboratory parameters and comorbidities of COVID-19 patients who were hospitalized, distinguishing between those who did not survive and those who recovered.

Finding	Outcome of Hospitalized Patients with COVID-19		*p*-Value
	Patients Who Did Not Survive (*n* = 27)	Patients Who Recovered from COVID-19 (*n* = 25)	
Type 2 diabetes mellitus *n* (%)	10 (37.0)	9 (36.0)	0.938
Hypertension *n* (%)	17 (62.96)	7 (28.0)	0.012 *
Obesity *n* (%)	7 (25.9)	2 (8.0)	0.143
Glucose on admission (mg/dL)	178.92 ± 75.80	192.50 ± 144.72	0.389
Glucose at discharge (mg/dL)	184.07 ± 103.74	140.42 ± 65.38	0.171
Total cholesterol (mg/dL)	161.66 ± 43.78	160.35 ± 21.92	0.946
Triglycerides (mg/dL)	326.80 ± 334.31	230.73 ± 127.39	0.943
Uric acid (mg/dL)	6.020 ± 2.35	8.40 ± 3.91	0.292
Serum creatinine (mg/dL)	2.022 ± 3.22	1.44 ± 3.17	0.1
Urea (mg/dL)	57.48 ± 45.36	60.86 ± 51.55	0.788
Blood urea nitrogen (mg/dL)	30.08 ± 15.62	28.68 ± 23.92	0.236
Erythrocyte Sedimentation Rate (mm/h)	21.53 ± 6.60	33.00 ± 21.21	0.231
Dimer D (ng/mL)	1.36 ± 1.24	2.60 ± 1.72	0.14
Lactate dehydrogenase (μ/L)	688.72 ± 272.70	551.75 ± 354.41	0.124
C Reactive Protein (mg/L)	73.62 ± 107.49	30.58 ± 15.09	0.852
Procalcitonin (ng/mL)	1.87 ± 2.48	0.35 ± 0.21	0.354
Hemoglobin (gr/dL)	12.32 ± 1.95	13.05 ± 2.90	0.353
Hematocrit (%)	39.28 ± 9.46	40.01 ± 8.45	0.837
Mean corpuscular volume (fL)	85.67 ± 8.15	88.13 ± 8.00	0.433
Mean corpuscular hemoglobin (pg/cell)	29.20 ± 2.64	28.54 ± 2.82	0.402
Mean corpuscular hemoglobin (g/dL)	33.63 ± 1.32	32.06 ± 1.67	0.015 *
Red cell distribution width (%)	14.22 ± 2.72	16.58 ± 9.40	0.640
Platelets (10^3^/μL)	205.23 ± 67.88	336.42 ± 157.30	0.003 *
Leukocytes (10^3^/μL)	13.10 ± 6.89	10.78 ± 4.11	0.474
Lymphocytes (%)	5.29 ± 3.61	16.36 ± 11.30	<0.001 *
Neutrophils (%)	83.07 ± 21.82	73.39 ± 19.83	0.006 *

Data are represented as frequency and percentages. * *p* < 0.05.

**Table 7:** Comparison of allele and genotype frequencies between recovery and death outcomes.

dbSNP ID	Genotype/Minor Allele	Patients Who Recovered from COVID-19 (*n* = 25)	Patients Who Did Not Survive (*n* = 27)	OR (95% CI)	*p*-Value
*RNASEL* rs486907	C/C	16 (64.0)	20 (74.1)	1	0.68
	C/T	7 (28.0)	6 (22.2)	0.69 (0.19–2.45)	
	T/T	2 (8.0)	1 (3.7)	0.40 (0.03–4.82)	
	Allele T	8 (20.0)	8 (15.0)	0.61 (0.23–1.67)	0.34
*OAS1* rs10774671	A/A	24 (96.0)	26 (96.3)	1	0.85
	A/G	1 (4.0)	1 (3.7)	0.92 (0.05–15.6)	
	Allele G	1 (20.0)	1 (20.0)	0.93 (0.05–15.2)	0.95
*OAS2* rs1293767	G/G	20 (80.0)	22 (81.5)	1	0.89
	C/G	5 (20.0)	5 (18.5)	0.91 (0.23–3.61)	
	Allele C	4 (10.0)	4 (8.0)	0.91 (0.25–3.38)	0.89
*OAS3* rs2285932	C/C	19 (76.0)	22 (81.5)	1	0.63
	C/T	6 (24.0)	5 (18.5)	0.72 (0.19–2.74)	
	Allele T	6 (12.0)	5 (8.0)	0.75 (0.21–2.62)	0.65

Abbreviations: CI, confidence interval; OR, odds ratio. Data are represented as frequency and percentages.

**Table 8:** Analysis of haplotype frequencies between symptomatic and asymptomatic groups.

	*RNASEL*	*OAS1*	*OAS2*	*OAS3*	Freq	OR (95% CI)	*p*-Value
1	C	A	G	C	0.6794	1	---
2	T	A	G	C	0.1855	1.83 (0.77–4.35)	0.18
3	C	A	C	T	0.1035	0.43 (0.16–1.19)	0.11
4	T	A	C	T	0.0076	0.00 (−Inf–Inf)	1
rare	C	G	G	C	0.024	1.08 (0.15–7.87)	0.94
Global haplotype association *p*-value							0.17

Abbreviations: OR, odds ratio.

**Table 9:** Analysis of haplotype frequencies between non-hospitalized and hospitalized patients.

	*RNASEL*	*OAS1*	*OAS2*	*OAS3*	Freq	OR (95% CI)	*p*-Value
1	C	A	G	C	0.6877	1	---
2	T	A	G	C	0.1775	1.08 (0.53–2.19)	0.83
3	C	A	C	T	0.0863	0.98 (0.34–2.85)	0.97
4	T	A	C	T	0.0244	0.00 (−Inf–Inf)	1
rare	T	G	C	T	0.024	1.09 × 10^9^ (1.085 × 10^9^–1.0851 × 10^9^)	<0.0001 *
Global haplotype association *p*-value							0.26

Abbreviations: OR, odds ratio. * *p* < 0.05.

**Table 10:** Analysis of haplotype frequencies between patients who survived and those who died because of COVID-19.

	*RNASEL*	*OAS1*	*OAS2*	*OAS3*	Freq	OR (95% CI)	*p*-Value
1	C	A	G	C	0.701	1	---
2	T	A	G	C	0.182	0.59 (0.22–1.56)	0.29
3	C	A	C	T	0.086	0.58 (0.13–2.57)	0.48
4	C	G	G	C	0.009	0.00 (−Inf–Inf)	1
5	C	A	G	T	0.009	0.00 (−Inf–Inf)	1
rare	C	G	C	T	0.009	1.03 × 10^20^ (1.031 × 10^20^–1.0309 × 10^20^)	<0.0001 *
Global haplotype association *p*-value							0.36

Abbreviations: OR, odds ratio. * *p* < 0.05.

**Table 11:** Summary of association studies between gene variants of the OAS/RNase L pathway and COVID-19.

Author/Year	Population	Study Design (*n*)	Gene Variants Evaluated	Findings
Kozak K, et al., 2023 [36].	Ukrainian	Case-control (*n* = 75 children: 30 with mild or moderate disease, 30 with severe COVID-19 and multisystem inflammatory syndrome (MIS-C), and 15 without COVID-19).	*ACE2* rs2074192, *IFNAR2* rs2236757, *TYK2* rs2304256, *OAS1* rs10774671, *OAS3* rs10735079, *CD40* rs4813003, *FCGR2A* rs1801274, *CASP3* rs113420705.	*ACE2* rs2074192-T, *IFNAR2* rs2236757-A, *OAS1* rs10774671-A, *CD40* rs4813003-C, *CASP3* rs113420705-C and male sex contribute to severe COVID-19 course and MIS-C in 85.6% of cases.
Skerenova M, et al., 2024 [34].	Slovakian	Case-control (*n* = 202; 139 COVID-19 cases and 63 controls).	17 single nucleotide variants (SNVs) in 11 genes: *CD209*, *DPP9*, *OAS1* (including rs10774671), *OAS3*, *TYK2*, *IFNAR2*, *CCHCR1*, *HLA-G*, *NOTCH4*, *THBS3*, *LZTFL1.*	There was an influence of LZTFL1 and *OAS1/OAS3* genetic variants on the severity of COVID-19. The *OAS1/OAS3* haplotype ‘GTTG’ carrying a functional allele G of splice-acceptor variant rs10774671 manifested its protective function in the Delta pandemic wave.
Udomsinprasert W, et al., 2023 [37].	Thai	Case-control (*n* = 260 patients with COVID-19; 239 mild and 21 severe COVID-19).	37 candidate genetic variants, including *OAS3* rs10735079.	*LZTFL1* rs10490770, rs11385942, rs17713054, *NADSYN1* rs12785878, *PLXNA4* rs1424597, *IL10* rs1800896, *ACE2* rs2285666, *PEDS1* rs6020298, *IL10RB* rs8178562 were related to long-term symptoms, incidence of long COVID. *OAS3* rs10735079 was not associated to severity, long-term symptoms or long COVID occurrence.
Rüter J, et al., 2022 [38].	German	Case-control, (*n* = 217: 123 COVID-19 cases and 94 controls).	30 single nucleotide variants including *OAS1* rs2660 and rs1131454, and *OAS3* rs10735079.	Genetic variants in *LZTFL1, APOE, ABO, FURIN, NOTCH4*, *CCL2*, *DPP9, IL6, OAS1* were associated with at least one of the phenotypes “susceptibility to infection”, “hospitalization”, or “severity”. LZTFL1 rs73064425 with hospitalization and severity, whereas rs1024611 near *CCL2* and rs1131454 in *OAS1* with susceptibility.
Lee D, et al., 2023 [33].	European, African, Asian, and American	Cohort (*n* = 558 patients with MIS-C, 1288 children and adults with asymptomatic or paucisymptomatic SARS-CoV-2 infection.	Whole-exome/whole-genome sequencing which included *OAS1*, *OAS2* and *RNASEL*.	Identification of autosomal recessive deficiencies of *OAS1*, *OAS2*, or *RNASEL* (*OAS1* p.R47*, *OAS2* p.R535Q, p.Q258L, and p.V290I, and *RNASEL* p.G59S + E265*) as genetic etiologies of MIS-C in 5 children. In these patients, MIS-C may result from an excessive immune cell response to SARSCoV-2. *RNASE*L/*OAS1-2* genetic deficiency resulted in the immunological and clinical phenotype of MIS-C.
Banday AR, et al., 2021 [39].	Patients from COVNET project (U.S. and Canada)	Case-control (*n* = 601 non-hospitalized vs. 954 hospitalized patients with COVID-19).	19Kb-haplotype that included 76 *OAS1* variants.	Rs10774671 and rs1131454 affect splicing and nonsense-mediated decay of *OAS1*. Genetically-regulated loss of *OAS1* expression contributes to impaired spontaneous clearance of SARS-CoV-2 and elevated risk of hospitalization for COVID-19.
Tanimine N, et al., 2021 [40].	Japanese	Cohort (*n* = 230 patients with COVID-19; 202 non-severe and 28 severe).	34 polymorphisms from 14 distinct candidate genes, including *OAS1* rs1131454, rs2660, and rs10774671.	Rs1131454 (*OAS1*) and rs1143627 (*IL-1B*) were associated with the severity of SARS-CoV-2 (adjusted odds ratio = 7.1 and 4.6 in the dominant model, respectively).
Dieter C, et al., 2023 [41].	Brazilian	Nested cohort case-control study (*n* = 694 with COVID-19: 414 critically ill patients with severe COVID-19 and 280 non-critically ill; 469 survivors and 183 non-survivors).	*ACE1* rs1799752, *ACE2* rs2285666, *DPP9* rs2109069, *IFIH1* rs1990760, *IFNAR2* rs2236757, *IFNL4* rs368234815, *TLR3* rs3775291, *TMPRSS2* rs12329760, *TYK2* rs2304256.	There was an association of rs1799752/*ACE1*, rs1990760/*IFIH1*, rs2236757/*IFNAR2*, rs12329760/*TMPRSS2*, and rs2304256/*TYK2* with worse COVID-19 outcomes, especially among female and non-white patients.
He J, et al., 2006. [14].	Chinese	Case-control (*n* = 130).	*OAS1* rs2660, *Mx -88* G/T (rs2071430).	SNPs in the *OAS1 3*′-UTR and MxA promoter region appear associated with host susceptibility to SARS.
Perez-Favila A, et al., 2024. This study.	Mexican	Cross-sectional, case-control (*n* = 104 patients with SARS-CoV-2 infection: 34 asymptomatic COVID-19 and 70 symptomatic COVID-19 cases.	*RNASEL* rs486907, *OAS1* rs10774671, *OAS2* rs1293767, and *OAS3* rs2285932.	Patients with the allele C of the *OAS2* gene rs1293767 (OR: 0.36, 95% CI: 0.15–0.83; *p* = 0.014;) and allele T of the *OAS3* gene rs2285932 (OR: 0.39, 95% CI: 0.2–0.023; *p* = 0.023) had lower susceptibility to developing symptomatic COVID-19.

## Data Availability

Dataset available on request from the authors.
